# 

**Published:** 1875-11

**Authors:** 


ESTABLISHED IN 1855.

Volume XIII. | NOVEMBER—1875. | Number 8.

S.G.O. INDEXED

### ATLANTA

### Medical and Surgical Journal.

MONTHLY: THREE DOLLARS PER ANNUM, IN ADVANCE.

EDITORS:

ROBERT BATTEY, M.D.,

AND

W. F. WESTMORELAND, M.D.

*PAX ET SCIENTIA, SED VERITAS SINE TIMORE.*

ATLANTA, GEORGIA:

*DUNLOP & DICKSON, BOOK AND JOB PRINTERS.*

1875.

